# The Cryoprotectant Effects of Safflower Polysaccharides on the Quality of Frozen–Thawed Boar Sperm

**DOI:** 10.3390/ani15060843

**Published:** 2025-03-15

**Authors:** Jingchun Li, Yingying Dong, Hechuan Wang, Qun Zhang, Qing Guo, Yanbing Li

**Affiliations:** College of Animal Science and Veterinary Medicine, Heilongjiang Bayi Agricultural University, Daqing 163319, China; dyy19980906@163.com (Y.D.); wanghechuan1999@outlook.com (H.W.); zzqdec@163.com (Q.Z.); qguo89@126.com (Q.G.); liyanbing929@163.com (Y.L.)

**Keywords:** boar sperm, safflower polysaccharides, cryopreservation, antioxidant

## Abstract

The low resistance of boar sperm to cryopreservation dictates that adding antioxidants and energetic substances to the diluent to improve sperm quality is necessary. This study is designed to assess the impact of various concentrations of safflower polysaccharides (SPSs; 0, 0.5, 1.0, 1.5, and 2.0 g/L) on the quality of boar sperm following freezing and thawing. The results of the study showed that the supplementation of 1.5 g/L SPS significantly enhanced the motility, average path velocity, straight-line velocity, curvilinear velocity, beat cross frequency, acrosome integrity, plasma membrane integrity, mitochondrial activity, and DNA integrity compared with the control group (*p* < 0.05). In addition, the supplementation of 1.5 g/L SPS significantly enhanced the total antioxidant capacity, superoxide dismutase activity, glutathione peroxidase activity, and catalase activity while significantly decreasing malondialdehyde and hydrogen peroxide content (*p* < 0.05). Therefore, the supplement SPS has potentially positive implications for improving the quality of cryopreserved boar sperm, and the recommended concentration is 1.5 g/L SPS.

## Simple Summary

During the process of cryopreservation of boar sperm, a large amount of reactive oxygen species is generated, leading to oxidative stress in the sperm and reducing the quality of sperm after thawing. Research has found that SPS has significant antioxidant activity. This study aims to investigate the effects of SPS on the quality and antioxidant capacity of thawed semen. The results indicate that SPS can significantly enhance sperm motility and increase the integrity of the acrosome, plasma membrane, and DNA. At the same time, it significantly enhances the antioxidant capacity of sperm, reduces oxidative stress in sperm, and improves the quality of thawed boar semen. In this study, the addition of 1.5 g/L SPS is considered the optimal concentration.

## 1. Introduction

Artificial insemination (AI) made its big breakthrough with the advent of stored liquid sperm in boars. Effective cryopreserved boar sperm enables the application of valuable germplasm resources to more females while reducing the transmission and occurrence of reproductive diseases [1]. Among the various in vitro sperm preservation techniques, cryopreservation has the advantages of almost unlimited preservation time and long-distance transportation compared to liquid preservation, but it also faces many challenges, such as reduced sperm viability, acrosome injury, and plasma membrane damage [1,2].

Boar spermatozoa generally exhibit lower quality compared to the frozen sperm of other livestock species. This is primarily attributed to the high content of unsaturated phospholipids and the relatively low cholesterol levels present in the cell membrane [3,4]. The composition of this membrane directly affects the quality of frozen boar sperm. Additionally, sperm cells inevitably generate reactive oxygen species (ROS) during metabolism. Under normal conditions, the intracellular antioxidant system can quickly neutralize ROS. In boar spermatozoa, moderate ROS fluctuations regulate protein kinases and phosphatases, which are essential for sperm capacitation. However, the disulfide bonds in sperm proteins are partially broken, and elevated ROS levels can also lead to the decondensation of sperm pronuclei following fertilization [5]. Unfortunately, during cryopreservation, ROS overproduction often surpasses the antioxidant defense mechanisms’ capacity, leading to oxidative damage in spermatozoa [6]. This oxidative stress leads to a decline in motility and damage to the axoneme of sperm and is accompanied by an increase in morphological abnormalities, particularly in the mid-piece, detrimental to capacitation and the acrosome reaction [6]. In recent years, polysaccharide compounds have been recognized for their significant protective effects in boar sperm cryopreservation. Various polysaccharides, including *Epimedium polysaccharide* [7], *Poria cocos* mushroom polysaccharides [8], *Salvia miltiorrhiza* [9], *Rhodiola rosea* [10], and Astragalus [11], have been studied for their potential to improve the quality of cryopreserved boar sperm.

The safflower (*Carthamus tinctorius*) is a traditional herbaceous crop widely cultivated in Afghanistan, India, Mexico, and other regions [12]. An important water-soluble polysaccharide extracted from safflower, known as safflower polysaccharide (SPS), possesses immunomodulatory and antioxidant properties [13]. The antioxidant capacity of SPS has been confirmed by several tests. Wan studied the antioxidant properties of SPS in H22 tumor-bearing mice, with the findings showing an increase in the activity of critical antioxidant enzymes (GR, GSH-PX, and SOD), as well as a decrease in the ROS and MDA levels in the high-dose group [14]. These findings suggest that SPS is effective in inhibiting ROS production and can partially delay the depletion of SOD in the body, thus offering protection against lipid peroxidation. Furthermore, purified polysaccharides (SPS2, SPS3) have demonstrated antioxidant properties in vitro tests [15,16]. According to our knowledge, there is currently no relevant research on the impact of SPS on the quality of frozen boar sperm. The experiment aims to investigate the effect of adding SPS to the cryopreservation extender on the quality of boar sperm after freezing and thawing.

## 2. Materials and Methods

### 2.1. Ethics Approval

All experiments were approved by the Ethics Committee of Heilongjiang Bayi Agricultural University (License No.: DWKJXY2023058). This research was conducted strictly following relevant guidelines and under supervision.

### 2.2. Chemicals and Sources

All the chemicals used in the study were obtained from Sigma–Aldrich (St. Louis, MO, USA), unless otherwise specified. SPS (BR, 98%) was sourced from Yita Biology (Beijing, China).

According to the report by Li et al. [17], the thawing agent and the cryopreservation extending agents I and II were configured. Cryopreservation extender I comprised 102.25 mmol/L lactose, 1 million U/L streptomycin, 194.27 mmol/L glucose, and 200 mL/L egg yolk. The pH was 7.2 and the osmolarity of the cryopreservation media was 270 mOsmol/kg. The reagent was prepared on a sterile bench. Subsequently, the cryopreservation extender was centrifuged (Sigma Laborzentrifugen, Osterode am Harz, Germany) at 4 °C for 10 min at 9000× *g*, and the supernatant after centrifugation was stored at 4 °C.

Glycerin 7% (*v*/*v*) was added to cryopreservation extender I to yield cryopreservation extender II. SPS at concentrations of 0, 0.5, 1.0, 1.5, and 2.0 g/L was mixed into cryopreservation extenders I and II. The sperm thawing agent for cryopreservation (pH 7.4 ± 0.1) contained 2.47 mmol/L citric acid, 34.62 mmol/L ethylenediamine, 23.17 mmol/L caffeine, and 138.77 mmol/L glucose.

### 2.3. Sperm Collection, Cryopreservation, and Thawing

Semen was gloved-hand method collected four times from eight Duroc boars aged 2–3, and was collected once a week at a commercial boar station (Jingyu Animal Husbandry, Daqing, China). The boars were kept in separate pens with natural light and provided sufficient complete feed for breeding boars (Shenyang Hefeng Animal Husbandry, Shenyang, China) and water. Semen was collected using a disposable semen collection bottle (60 mL) and placed in a 37 °C incubator (Summer New Livestock Special Insulation Box, 10 L). The collected semen was then delivered to the laboratory within half an hour for preliminary quality assessment using a fully automated sperm analyzer (CASA; Songjing Tianlun Biotechnology, Nanning, China). Semen samples that are odorless, meet color standards, and have sperm viability ≥90% were selected.

The semen samples were centrifuged (CENCE Laboratory Instruments, Changsha, China) at 290× *g* for 10 min and the supernatants were discarded. Cryopreservation extender I was then used to adjust the sperm concentration to 4 × 10^8^/mL, and it was placed in a 17 °C incubator to equilibrate for 20 min. Afterwards, cryopreservation extender II was used to further adjust the sperm concentration to 2 × 10^8^/mL, and the mixture was equilibrated at 4 °C for 50 min. The resulting extended sperm samples were used to fill 0.5 mL sperm straws. A total of 120 straws were allocated for each sampling day, such that an average of 24 straws were prepared per treatment group.

The procedures used for freezing and thawing the sperm were those described by Gadea et al. [18] but were appropriately modified. The sperm straws were placed in a cryogenic programmer (CryoLogic, Melbourne, Australia), and the freezing procedure was as follows: The temperature was reduced from 4 °C to −5 °C at a rate of −6 °C/min, then it was held for 60 s. Subsequently, the temperature was reduced to −120 °C at the same rate, and then held at this temperature for 60 s. After freezing, the straws were quickly transferred to liquid nitrogen, where they were stored for 30 days.

The process of sperm thawing is as follows: quickly remove the frozen sperm straw from liquid nitrogen, revive it at 50 °C for 15 s, mix 300 µL of thawing agent with the thawed sperm, and incubate at 37 °C in preparation for subsequent testing.

### 2.4. Determination of Sperm Quality and Kinetic Parameters

According to the method described by Tvrdá et al. [19], CASA is used to detect sperm kinetic parameters. A total of 10 µL of thawed sperm (use the thawing agent diluted to 1 × 10^8^/mL during thawing) was placed onto a slide (Jiangsu Feizhou Glass-Plastic—Sailboat Brand Glass Slides), pre-warmed to 37 °C, and covered with a coverslip. Next, five fields under a 200× optical microscope (ML-800 II; Songjing Tianlun Biotechnology, Nanning, China) were examined, ensuring that there were at least 150 sperm in each field. The quality and motility parameters of the sperm were then analyzed using the CASA sperm analysis system. The parameters measured included the average path velocity (VAP, µm/s), beat cross frequency (BCF, Hz/s), straight-line velocity (VSL, µm/s), and curvilinear velocity (VCL, µm/s). The sperm viability is defined as the proportion of viable sperm cells in a semen sample [20]. In a semen sample, the total proportion of all motile sperm is referred to as sperm motility [21]. The rate of abnormal sperm refers to the percentage of abnormal sperm among the total sperm. Abnormal sperm includes defects such as biflagellate, excessive curvature, decapitation, taillessness, and bifid heads [22]. The experiment was repeated four times.

### 2.5. Determination of Sperm Acrosome Integrity and Plasma Membrane Integrity

The integrity of sperm acrosome was assessed using the fluorescein isothiocyanate-conjugated peanut agglutinin (FITC-PNA) staining method, as reported by Sun et al. [23]. Briefly, 20 µL of each sperm sample was used to prepare thin smears, which were air-dried, fixed in anhydrous methanol for 10 min, and stained with 20 µL fluorescein isothiocyanate-conjugated peanut agglutinin (FITC-PNA). The stained smears were then incubated in the dark for 20 min, washed with HEPES, and air dried. The slides were examined using a fluorescence microscope (EVOS FL). Five fields with at least 100 sperm per field were observed for each sample. The experiment was repeated four times.

According to the method described by Kou et al. [24], SYBR-14 and PI staining were used to detect sperm mitochondrial activity. Briefly, 250 µL of each sample was centrifuged for 5 min at 290× *g* and the supernatant was discarded. Subsequently, 125 µL of PBS was be used to resuspend the precipitated sperm, and the samples were then each combined with 5 µL SYBR-14 solution and 5 µL PI solution and incubated in an incubator 37 °C for 15 min in the dark. Next, 10 µL samples were pipetted onto slides for examination using a fluorescence microscope. For each sample, five fields were examined, with at least 100 sperm in each field. The experiment was repeated four times.

### 2.6. Determination of Sperm Mitochondrial Activity and DNA Integrity

According to the method described by Wa et al. [25], JC-1 staining was used to detect sperm mitochondrial activity. In brief, 250 µL of each sample was centrifuged (5 min, 290× *g*) and the supernatant was discarded. A total of 125 µL of PBS was used to resuspend the precipitated sperm, then the sample was mixed with 10 µL JC-1 and incubated at 37 °C for 30 min. A 10 μL sample was taken and placed on a slide for examination under a fluorescence microscope. Five fields with at least 100 sperm per field were then examined. The experiment was repeated four times.

According to the methods described by Ammar et al. [26] and Nur Karakus et al. [27], the integrity of sperm DNA was detected using acridine orange (AO) staining. Briefly, 20 µL of sperm samples were used to prepare thin smears, which were air-dried, fixed with anhydrous methanol for 10 min, and stained with 20 µL Ao. The stained slides were incubated in a dark environment at 37 °C for 30 min, rinsed with HEPES, and then allowed to air-dry naturally. The slides were examined using a fluorescence microscope. Five regions were selected for each group of samples, with at least 100 sperm in each region. The experiment was repeated four times.

### 2.7. Determination of Sperm Antioxidant Capacity

According to the report by Shi et al. [28], the antioxidant capacity of sperm was tested using a kit following the manufacturer’s instructions (Jiancheng Institute of Bioengineering, Nanjing, China). The total antioxidant capacity (T-AOC), malondialdehyde (MDA) content, glutathione peroxidase (GSH-Px) activity, hydrogen peroxide (H_2_O_2_) content, catalase (CAT) activity, and superoxide dismutase (SOD) activity were measured. Sample preparation was performed according to the instructions provided in the kits. A T-AOC kit (model: A015-1-2; colorimetric method) was used to measure the T-AOC capacity, and the optical density (OD) value was assessed at 530 nm using a spectrophotometer. An MDA kit (model: A003-1-2; thiobarbituric acid (TBA) method) was used to measure the MDA content and the OD value was measured at 532 nm using the spectrophotometer. The GSH-Px activity was determined using a GSH-Px assay kit (model: A005-1-2; colorimetric method), and the OD value was measured at 532 nm using the spectrophotometer. The H_2_O_2_ content was determined using an H_2_O_2_ assay kit (model: A064-1-1; colorimetric method), and the OD value was measured at 532 nm using the spectrophotometer. The CAT activity was determined using a CAT kit (model: A007-1-1; ammonium molybdate method), and the OD value was measured at 405 nm using the spectrophotometer. SOD activity was determined using a SOD assay kit (model: A001-3-2; WTS-1 method), and the OD value was measured at 405 nm using a microplate reader (Bio-Rad, Hercules, CA, USA). The experiment was repeated four times.

### 2.8. Statistical Analysis

All results are expressed as values ± SD. Data analysis was conducted using SPSS 26 (IBM, New York, NY, USA). The analysis focused on sperm motility and motion parameters (VCL, VSL, VAP, ALH, and LIN), as well as the assessment of sperm acrosome, plasma membrane, DNA integrity, and mitochondrial activity. Both a one-way ANOVA and Duncan’s multiple range test were conducted on the data to determine significant differences among all parameters and statistical significance is defined as *p* < 0.05.

## 3. Results

### 3.1. Effects of SPS on Boar Sperm Quality and Kinetic Parameters

Table 1 presents the descriptive statistics of boar sperm quality following freeze-thawing.

Relative to the control group, supplementing with various concentrations of safflower polysaccharides improved the motility and viability of thawed sperm while decreasing the incidence of sperm abnormality (*p* < 0.05). Among the groups, the sperm vitality and motility in the 1.5 g/L SPS group were significantly superior to those in the other experimental groups, while the abnormality rate was markedly lower compared to the other groups (*p* < 0.05). In addition, sperm viability and motility were significantly increased in the 2.0 g/L SPS group compared to the 0.5 and 1.0 g/L SPS addition groups (*p* < 0.05), and abnormality rates were significantly reduced compared to the 0.5 and 1.0 g/L SPS groups (*p* < 0.05).

Table 2 presents the descriptive statistics of boar sperm kinematic parameters following freeze-thawing.

Among the groups, the sperm VAP, VSL, and VCL of the 1.5 g/L SPS group were significantly better than those of the other experimental groups (*p* < 0.05). In addition, sperm VAP, VSL, and VCL were significantly increased in the 2.0 g/L SPS group compared to the 0.5 and 1.0 g/L SPS groups (*p* < 0.05), meanwhile there were no significant statistical differences between the 0.5 and 1.0 g/L SPS groups (*p* > 0.05). The BCF of sperm in the 1.5 g/L SPS group was significantly higher than that in the 0 g/L SPS group (*p* < 0.05), and the BCFs in the 1.0 and 2.0 g/L SPS groups were both significantly higher than that in the 0.5 g/L SPS group (*p* < 0.05).

### 3.2. Effects of SPS on Acrosome Integrity and Plasma Membrane Integrity of Boar Sperm

**Figure 1:**
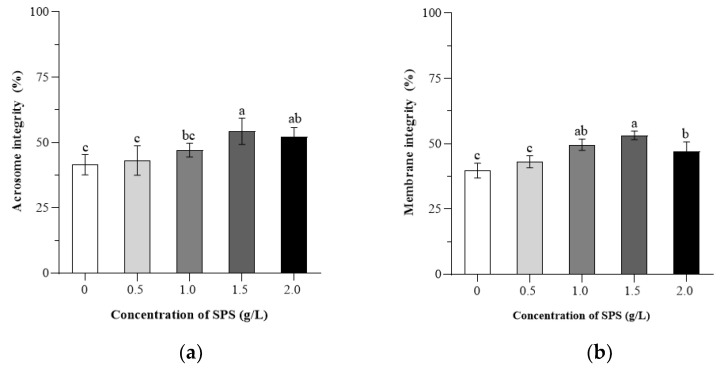
Effect of SPS on acrosome integrity (**a**) and membrane integrity (**b**) in cryopreserved boar sperm. Bars with different letters denote significant differences (*p* < 0.05) among groups.

Figure 1 presents the descriptive statistics of boar sperm acrosome integrity and plasma membrane integrity following freeze-thawing.

In this study, a significant increase in sperm acrosome integrity was found in the 1.0, 1.5, and 2.0 g/L SPS groups compared to the 0 g/L SPS group (*p* < 0.05). In addition, sperm acrosome integrity was reduced in the 0 and 0.5 g/L SPS groups compared to the 2.0 g/L SPS group (*p* > 0.05). The sperm high plasma membrane integrity rate of the 1.5 g/L SPS group was higher than that in 0, 0.5, and 2.0 g/L SPS groups (*p* < 0.05), and the rates in the 1.0 and 2.0 SPS groups were higher than those in the 0 and 0.5 g/L SPS groups (*p* < 0.05).

### 3.3. Effects of SPS on Mitochondrial Activity and DNA Integrity of Boar Sperm

**Figure 2:**
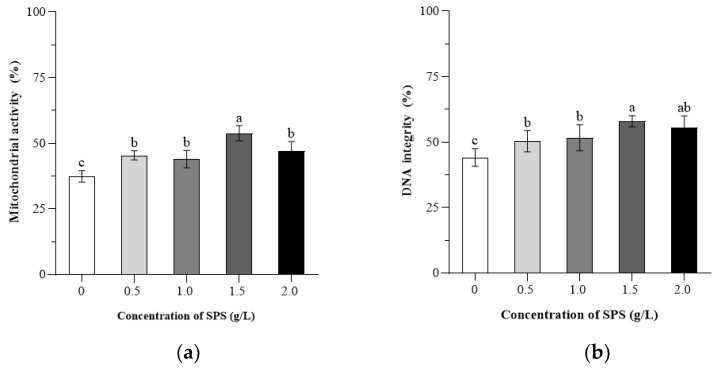
Effect of SPS on mitochondrial activity (**a**) and DNA integrity (**b**) of cryopreserved boar sperm. Bars with different letters denote significant differences (*p* < 0.05) among groups.

Figure 2 presents the descriptive statistics of boar sperm mitochondrial activity and DNA integrity following freeze-thawing.

The sperm high mitochondrial activity rates of the 0.5, 1.0, 1.5, and 2.0 g/L SPS groups were higher than those in the 0 g/L SPS group (*p* < 0.05), those in the 1.50 g/L SPS group were higher than those of the 0, 0.5, 1.0, and 2.0 g/L SPS groups (*p* < 0.05), and those of the 0.5, 1.0, and 2.0 SPS groups were higher than those in the 0 g/L SPS group (*p* < 0.05). Sperm high DNA integrity rates of the 0.5, 1.0, 1.5, and 2.0 g/L SPS groups were higher than those in the 0 g/L SPS group (*p* < 0.05), and the 1.5 g/L SPS group showed significantly higher DNA integrity compared to the 0, 0.5, and 1.0 g/L SPS groups (*p* < 0.05), while the 0.5 and 1.0 g/L SPS groups were significantly higher than the 0 g/L SPS group (*p* < 0.05). No significant differences were observed between the 0.5 and 1.0 g/L SPS groups (*p* > 0.05).

### 3.4. Effects of SPS on the Antioxidant Capacity of Boar Sperm

**Figure 3:**
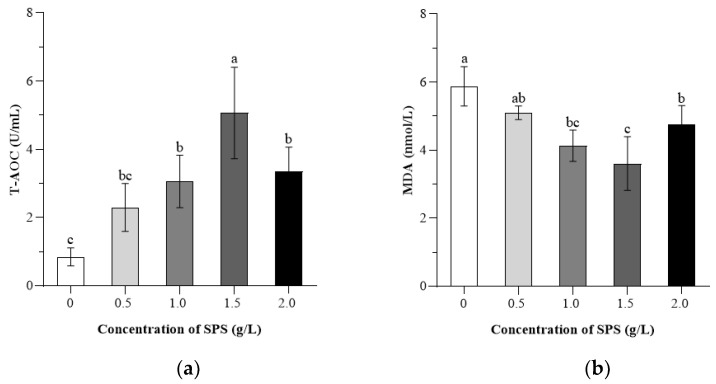

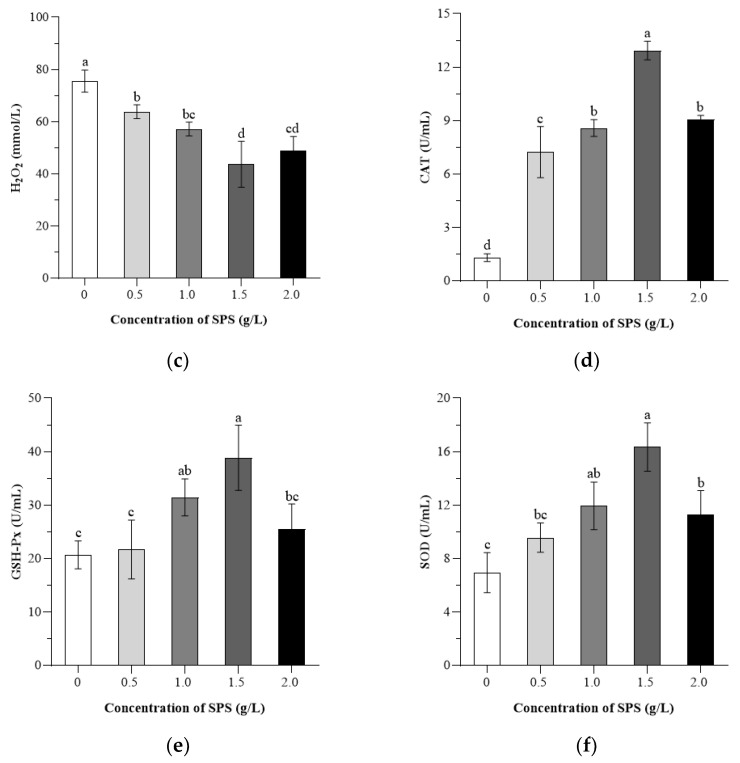
Effect of SPS on T-AOC (**a**), MDA content (**b**), H_2_O_2_ content (**c**), CAT activity (**d**), GSH-Px activity (**e**), and SOD activity (**f**) of cryopreserved boar sperm. Bars with different letters denote significant differences (*p* < 0.05) among groups.

Figure 3 presents the descriptive statistics of boar sperm antioxidant capacity following freeze-thawing.

The T-AOC in the 1.50 g/L SPS group was significantly greater than that in both the 1.0 and 2.0 g/L SPS group (*p* < 0.05). Both the 1.0 and 2.0 g/L SPS groups showed higher T-AOC levels compared to the 0 g/L SPS group (*p* < 0.05). In terms of MDA content, the 0 g/L SPS group is higher than those of the 1.0, 1.5, and 2.0 g/L SPS groups (*p* < 0.05). The MDA content of the 1.5 g/L SPS group is lower than those of the 1.0 and 2.0 g/L SPS groups (*p* < 0.05). The H_2_O_2_ content in the 0 g/L SPS group is higher than those in the 0.5, 1.0, 1.5, and 2.0 g/L SPS groups (*p* < 0.05), while the content in the 1.5 g/L SPS group is lower than those in the other SPS groups (*p* < 0.05).

The CAT activity in the 1.5 g/L SPS group is higher than those in the 0, 0.5, 1.0, and 2.0 g/L SPS groups (*p* < 0.05). The CAT activity in the 1.0 and 2.0 g/L SPS groups is higher than that in the 0.5 g/L SPS group (*p* < 0.05). The GSH-Px activity in the 1.5 g/L SPS group was higher compared to the 0, 0.5, and 2.0 g/L SPS groups (*p* < 0.05). The 1.0 g/L SPS group also showed higher GSH-Px activity than the 0 and 0.5 g/L SPS groups (*p* < 0.05). Regarding SOD activity, the 1.5 g/L SPS group had higher activity than the 0, 0.5, and 2.0 g/L SPS groups (*p* < 0.05). Both the 1.0 and 2.0 g/L SPS groups exhibited higher SOD activity than the 0 g/L SPS group (*p* < 0.05).

## 4. Discussion

During the freezing process, sperm produces a large amount of reactive oxygen species (ROS), which leads to oxidative stress in sperm and causes irreversible damage. This damage impacts the quality of semen preservation and hinders the advancement of frozen semen [29]. Since cryopreservation technology was applied to the cryopreservation of boar sperm, adding various antioxidants, membrane protectants, and energy substances to the extender has gradually become a necessary protocol to improve its preservation quality [30]. Therefore, in this study and according to our previous knowledge, this could be the first time evaluation of the effects of SPSs added to the cryopreservation extender on the quality of frozen sperm in boars.

SPS is a water-soluble polysaccharide extracted from safflower, and due to its anti-inflammatory and antioxidant properties, it is widely regarded as a potentially effective drug for the prevention and treatment of various diseases [6,31]. The research conducted by Cui et al. [32] indicates that the use of SPSs at concentrations of 50 and 100 μg/mL during the process of osteoblast necrosis induced by dexamethasone can lead to enhanced cell viability and reduced apoptosis. However, as far as we know, there has not yet been any research on the impact of safflower polysaccharides on the quality of frozen semen in boars. The direct indicator of sperm quality is the motility of the sperm [33]. A high motility is crucial for sperm to travel from the reproductive tract to the ampulla of the fallopian tube and achieve fertilization [34]. Research has found that in male semen, the fertilization ability of sperm is directly related to its VAP, VSL, and VCL, and there is a strong correlation between BCF and sperm quality [35,36]. In ostrich sperm, VAP and VSL are considered the most important and reliable parameters for predicting fertility [37]. In this study, the VSL and VAP values of sperm in the 1.50 g/L SPS group were also relatively high. Furthermore, Byrne et al. [38] showed that supplementation of rumen-protected safflower to bulls did not improve their sperm volume and progressive linear motility. However, Nasiri et al. [39] found that safflower seed oil pre-treatment was effective in alleviating the level of sperm motility and sperm count decline in a diabetic model mice. Although there is a lack of studies on safflower polysaccharide in frozen semen of other animals, this study found that adding different concentrations of safflower polysaccharide during the freezing process significantly improved the quality and kinetic parameters of frozen boar semen compared to the 0 g/L SPS group, which is similar to the aforementioned research results.

Studies show that the preservation of sperm plasma membrane integrity is essential for the acrosome reaction and successful fertilization [40]. Boar sperm is highly sensitive to cold shock, which is attributed to its membrane characteristics: it contains a high proportion of polyunsaturated fatty acids (PUFA) whilst having a relatively low cholesterol content [41]. The cryopreserved process promotes the elevated level of lipid peroxidation in boar sperm, which further leads to membrane degeneration and damage [42]. Furthermore, the acrosomal enzymes present in the acrosome are key factors in promoting the acrosome reaction of sperm in the female reproductive tract. During the freezing and preservation process, partial or complete loss of acrosomal enzymes may lead to a decrease in sperm fertilization ability [43,44]. The sperm of boars has a relatively weak tolerance to low-temperature environments, and drastic changes in the external environment during the freezing preservation period may cause damage to DNA integrity [45]. Research has found that a decrease in the integrity of sperm DNA has a negative impact on fertility [46]. In this study, we further found that the 1.5 g/L SPS group significantly improved the integrity of frozen boar sperm DNA, acrosome, and plasma membrane compared to the 0 g/L SPS group. Although polysaccharides, as macromolecules, cannot penetrate the plasma membrane to balance the adverse effects of osmotic pressure and other factors during cryopreservation, they can surround the outer layer of the plasma membrane to help resist the harmful effects of ice crystals during the danger zone of cryopreservation [47]. This may be a potential mechanism by which SPS enhances the quality of frozen boar sperm membranes and acrosome integrity.

Under normal circumstances, ROS in sperm are maintained at physiological concentrations by antioxidant and non-antioxidant enzyme systems. However, during the cryopreservation process, this balance may be disrupted by drastic temperature changes [48]. Excessive ROS can damage the PUFAs in the sperm plasma membrane of boars, causing changes in membrane fluidity and permeability and leading to sperm oxidative stress, which ultimately affects sperm quality after thawing [49]. Polysaccharides are considered to exert their antioxidant effects primarily by influencing the NF-κB pathway and the Nrf2/Keap1 ARE signaling pathway [50]. Studies have shown that polysaccharide intake can activate specific signaling pathways and increase the expression of antioxidant-related genes. These include enhanced activity or levels of enzymes such as CAT, GSH-Px, and SOD, as well as glutathione and glutathione reductase, which protect against cell damage caused by oxidative stress [51]. By analyzing the chemical structure of SPS, Lin et al. [52] found that it was mainly composed of glucose, galactose, and arabinose and exhibited relatively high antioxidant activity in vitro. Weng et al. [53] found that adding APS to thawed boar sperm reduces ROS levels and enhances SOD and CAT activity, whilst Zangishhi et al. [54] reported that adding kombu polysaccharides to the frozen diluent improves SOD activity and membrane integrity of frozen–thawed ram semen, whilst also reducing MDA production. In this study, the T-AOC capacity of thawed sperm, as well as the levels of MDA and H_2_O_2_, and the activities of CAT, SOD, and GSH-Px were detected. We found that the 1.5 g/L SPS group of sperm exhibited significant antioxidant properties compared to the 0 g/L SPS group, increased T-AOC capacity, CAT and SOD activity, which effectively prevented damage to boar sperm caused by ROS. This is similar to the results of the aforementioned polysaccharide antioxidants in the application of frozen semen from other animals. These results indicate that adding SPS to the frozen diluent can enhance the ability of thawed semen to resist oxidative stress.

## 5. Conclusions

According to our previous and current knowledge, the results of this study could be one of the first results represent the first verification of the effect of SPS on the cryopreservation of boar semen. The addition of 1.5 g/L SPS significantly improved the quality and antioxidant capacity of frozen boar sperm. Hopefully, this finding will provide important scientific evidence for the development of boar semen cryopreservation technology.

## Figures and Tables

**Table 1:** Effects of SPS on quality parameters of cryopreserved boar sperm.

Groups	Items		
	Viability (%)	Motility (%)	Abnormality Rate (%)
0 g/L SPS	72.33 ± 0.33 ^d^	62.65 ± 0.73 ^c^	22.52 ± 0.85 ^a^
0.5 g/L SPS	73.33 ± 0.04 ^c^	64.53 ± 1.03 ^c^	18.26 ± 0.91 ^b^
1.0 g/L SPS	73.77 ± 0.07 ^c^	69.27 ± 1.47 ^b^	14.87 ± 0.09 ^c^
1.5 g/L SPS	78.00 ± 0.37 ^a^	73.92 ± 2.04 ^a^	12.46 ± 0.13 ^d^
2.0 g/L SPS	76.83 ± 0.30 ^b^	70.70 ± 2.20 ^b^	12.81 ± 1.06 ^d^

^a–d^ Mean values with different superscripts within columns are significantly different at *p* < 0.05.

**Table 2:** Effects of SPS on kinetic parameters of cryopreserved boar sperm.

Groups	Items			
	VAP (μm/s)	VSL (μm/s)	VCL (μm/s)	BCF (Hz)
0 g/L SPS	18.22 ± 4.31 ^c^	37.95 ± 1.57 ^c^	26.69 ± 7.28 ^c^	4.26 ± 0.83 ^d^
0.5 g/L SPS	21.39 ± 5.73 ^bc^	42.82 ± 1.25 ^b^	34.98 ± 6.82 ^bc^	8.57 ± 0.57 ^c^
1.0 g/L SPS	25.31 ± 3.38 ^bc^	43.58 ± 0.98 ^b^	43.76 ± 6.48 ^b^	11.47 ± 1.21 ^b^
1.5 g/L SPS	35.35 ± 4.93 ^a^	49.77 ± 3.36 ^a^	55.22 ± 1.79 ^a^	15.64 ± 1.24 ^a^
2.0 g/L SPS	28.08 ± 1.38 ^ab^	48.91 ± 2.42 ^a^	54.03 ± 3.61 ^a^	12.99 ± 0.14 ^b^

^a–d^ Mean values with different superscripts within columns are significantly different at *p* < 0.05.

## Data Availability

The data in this study can be obtained from the corresponding author upon request.

## References

[1] Roca J., Hernández M., Carvajal G., Vázquez J.M., Martínez E.A. (2006). Factors Influencing Boar Sperm Cryosurvival. J. Anim. Sci..

[2] Waberski D., Riesenbeck A., Schulze M., Weitze K.F., Johnson L. (2019). Application of Preserved Boar sperm for Artificial Insemination: Past, Present and Future Challenges. Theriogenology.

[3] Basioura A., Tsakmakidis I.A., Martinez E.A., Roca J., Li J., Molina M.F., Theodoridis A., Boscos C.M., Parrilla I. (2020). Effect of Astaxanthin in Extenders on Sperm Quality and Functional Variables of Frozen-Thawed Boar sperm. Anim. Reprod. Sci..

[4] O’Brien E., García-Casado P., Castaño C., Toledano-Díaz A., Bóveda P., Santiago-Moreno J. (2021). Sperm Response to in Vitro Stress Conditions in Wild and Domestic Species Measured by Functional Variables and ROS Production. Front. Vet. Sci..

[5] Betarelli R.P., Rocco M., Yeste M., Fernández-Novell J.M., Placci A., Azevedo Pereira B., Castillo-Martín M., Estrada E., Peña A., Zangeronimo M.G. (2018). The Achievement of Boar Sperm in Vitro Capacitation is Related to an Increase of Disrupted Disulphide Bonds and Intracellular Reactive Oxygen Species levels. Andrology.

[6] Pezo F., Yeste M., Zambrano F., Uribe P., Risopatrón J., Sánchez R. (2021). Antioxidants and Their Effect on the Oxidative/Nitrosative Stress of Frozen-Thawed Boar Sperm. Cryobiology.

[7] Li Y., Wang H., Hu Z., Zhang G., Wen F., Xian M., Guo S., Zhang G., Zhang X., Hu J. (2025). Supplementation of *Epimedium polysaccharide* (EPS) improves goat semen characteristics following cryopreservation. Anim. Reprod. Sci..

[8] Zhou J., Zhang K., Gao J., Xu J., Wu C., He M., Zhang S., Zhang D., Dai J., Sun L. (2023). Effect of *Poria cocos* Mushroom Polysaccharides (PCPs) on the Quality and DNA Methylation of Cryopreserved Shanghai White Pig Spermatozoa. Cells.

[9] Shen T., Jiang Z.L., Liu H., Li Q.W. (2015). Effect of *Salvia miltiorrhiza* Polysaccharides on Boar Spermatozoa During Freezing-Thawing. Anim. Reprod. Sci..

[10] Yang S.M., Wang T., Wen D.G., Hou J.Q., Li H.B. (2016). Protective Effect of *Rhodiola rosea* Polysaccharides on Cryopreserved Boar Sperm. Carbohydr. Polym..

[11] Fu J., Yang Q., Li Y., Li P., Wang L., Li X. (2018). A mechanism by which Astragalus polysaccharide protects against ROS toxicity through inhibiting the protein dephosphorylation of boar sperm preserved at 4 °C. J Cell. Physiol..

[12] Qin Y., Fan K., Yimamu A., Zhan P., Lv L., Li G., Liu J., Hu Z., Yan X., Hu X. (2025). Integrated Genetic Diversity and Multi-Omics Analysis of Colour Formation in Safflower. Int. J. Mol. Sci..

[13] Li J.X., Xu D.Q., Cui D.X., Fu R.J., Niu Z.C., Liu W.J., Tang Y.P. (2025). Exploring the structure-activity relationship of Safflower polysaccharides: From the structural characteristics to biological function and therapeutic applications. J. Ethnopharmacol..

[14] Wan Y.F. (2016). The Effects of *Carthamus tinctorius* Polysaccharides on the Immunity Function and Antioxidant Function of H22 Tumor-Bearing Mice. J. North Pharm..

[15] Zou Y.F., Ren A.N., Yao M.M., Gu X.H. (2011). Effect of Macroporous Adsorptive Resins on Decoloration Technology of *Carthamus tinctorius* Polysaccharide. China Pharm..

[16] Hu Y. (2020). Structural Analysis and Antioxidant Activities of Polysaccharide Isolated from *Carthamus tinctorius* L.. Master’s Thesis.

[17] Li J., Wang H., Guo M., Li T., Zhang H., Zhang Q., Wang Q., Song Y., Feng H., Wei G. (2023). Exogenous Spermidine Effectively Improves the Quality of Cryopreserved Boar Sperm. Anim. Sci. J..

[18] Álvarez-Rodríguez M., Nieto-Cristobal H., de Mercado E. (2024). Bovine serum albumin inclusion in the thawing extender improves boar sperm membrane and acrosomal integrity. Reprod. Domest. Anim..

[19] Tvrdá E., Greifová H., Mackovich A., Hashim F., Lukáč N. (2018). Curcumin Offers Antioxidant Protection to Cryopreserved Bovine Semen. Czech J. Anim. Sci..

[20] Takeshima M., Gotoh A. (2024). Establishment of a rapid, cost-effective, and accurate method for assessing insect sperm viability. J. Insect. Physiol..

[21] Zuchowicz N., Daly J., Bouwmeester J., Lager C., Henley E.M., Nuñez Lendo C.I., Hagedorn M. (2021). Assessing coral sperm motility. Sci. Rep..

[22] Menkveld R. (2010). Clinical significance of the low normal sperm morphology value as proposed in the fifth edition of the WHO Laboratory Manual for the Examination and Processing of Human Semen. Asian J Androl..

[23] Sun L., Fan X., Zeng Y., Wang L., Zhu Z., Li R., Tian X., Wang Y., Lin Y., Wu D. (2020). Resveratrol Protects Boar Sperm in Vitro Via its Antioxidant Capacity. Zygote.

[24] Kou Z., Hu B., Li Y., Cai R., Gao L., Chu G., Yang G., Pang W. (2022). Boar Seminal Plasma Improves Sperm Quality by Enhancing its Antioxidant Capacity During Liquid Storage at 17 °C. Zygote.

[25] Prochowska S., Eberhardt M., Smalec B., Niżański W. (2024). In search of freezability predictors for feline spermatozoa-osmotic challenge tests and markers of sperm membrane structure. Anim Reprod Sci..

[26] Ammar O., Mehdi M., Muratori M. (2020). Teratozoospermia: Its Association with Sperm DNA Defects, Apoptotic Alterations, and Oxidative Stress. Andrology.

[27] Nur Karakus F., Bulgurcuoglu Kuran S., Solakoglu S. (2021). Effect of Curcumin on Sperm Parameters after the Cryopreservation. Eur. J. Obstet. Gynecol. Reprod. Biol..

[28] Shi L., Zhang Y., Huang X., Shi M., Sun D., Zhang Y., Li W., Jin T., Feng J., Xing J. (2022). Effects of Mitoquinone (MitoQ) Supplementation During Boar sperm Cryopreservation on Sperm Quality, Antioxidant Status and Mitochondrial Proteomics. Anim. Reprod. Sci..

[29] Wang M., Wu S., Yang B., Ye M., Tan J., Zan L., Yang W. (2023). Grape Seed Proanthocyanidins Improve the Quality of Fresh and Cryopreserved Semen in Bulls. Animals.

[30] Funahashi H. (2015). Methods for Improving in Vitro and in Vivo Boar Sperm Fertility. Reprod. Domest. Anim..

[31] Wu X., Cai X., Ai J., Zhang C., Liu N., Gao W. (2021). Extraction, Structures, Bioactivities and Structure-Function Analysis of the Polysaccharides from Safflower (*Carthamus tinctorius* L.). Front. Pharmacol..

[32] Cui D., Zhao D., Wang B., Liu B., Yang L., Xie H., Wang Z., Cheng L., Qiu X., Ma Z. (2018). Safflower (*Carthamus tinctorius* L.) Polysaccharide Attenuates Cellular Apoptosis in Steroid-Induced Avascular Necrosis of Femoral Head by Targeting Caspase-3-Dependent Signaling Pathway. Int. J. Biol. Macromol..

[33] Lewandowska E., Węsierski D., Mazur-Milecka M., Liss J., Jezierska A. (2023). Ensembling noisy segmentation masks of blurred sperm images. Comput. Biol. Med..

[34] Van de Hoek M., Rickard J.P., de Graaf S.P. (2022). Motility Assessment of Ram Spermatozoa. Biology.

[35] Hirano Y., Shibahara H., Obara H., Suzuki T., Takamizawa S., Yamaguchi C., Tsunoda H., Sato I. (2001). Relationships between Sperm Motility Characteristics Assessed by the Computer-Aided Sperm Analysis (CASA) and Fertilization Rates in Vitro. J. Assist. Reprod. Genet..

[36] Liu D.Y., Clarke G.N., Baker H.W. (1991). Relationship between Sperm Motility Assessed with the Hamilton-Thorn Motility Analyzer and Fertilization Rates in Vitro. J. Androl..

[37] Muvhali P.T., Bonato M., Malecki I.A., Cloete S.W.P. (2022). Mass Sperm Motility Is Correlated to Sperm Motility as Measured by Computer-Aided Sperm Analysis (CASA) Technology in Farmed Ostriches. Animals.

[38] Byrne C.J., Fair S., English A.M., Holden S.A., Dick J.R., Lonergan P., Kenny D.A. (2017). Dietary Polyunsaturated Fatty Acid Supplementation of Young Post-Pubertal Dairy Bulls Alters the Fatty Acid Composition of Seminal Plasma and Spermatozoa but has no Effect on sperm Volume or Sperm Quality. Theriogenology.

[39] Nasiri K., Akbari A., Nimrouzi M., Ruyvaran M., Mohamadian A. (2021). Safflower Seed Oil Improves Steroidogenesis and Spermatogenesis in Rats with Type II Diabetes Mellitus by Modulating the Genes Expression Involved in Steroidogenesis, Inflammation and Oxidative Stress. J. Ethnopharmacol..

[40] Ritagliati C., Baro Graf C., Stival C., Krapf D. (2018). Regulation mechanisms and implications of sperm membrane hyperpolarization. Mech. Dev..

[41] Wang S., Wang Q., Min L., Cao H., Adetunji A.O., Zhou K., Zhu Z. (2025). Sperm Pyrroloquinoline Quinone Improved Boar Sperm Quality via Maintaining Mitochondrial Function During Cryopreservation. Antioxidants.

[42] Almubarak A., Kim E., Yu I.J., Park H., Jeon Y. (2024). The Effect of κ-Carrageenan on Porcine Sperm Cryo-Survival. Animals.

[43] Pinart E., Yeste M., Bonet S. (2015). Acrosin Activity is a Good Predictor of Boar Sperm Freezability. Theriogenology.

[44] Ded L., Dostalova P., Zatecka E., Dorosh A., Komrskova K., Peknicova J. (2019). Fluorescent Analysis of Boar Sperm Capacitation Process in vitro. Reprod. Biol. Endocrinol..

[45] Alkmin D.V., Martinez-Alborcia M.J., Parrilla I., Vazquez J.M., Martinez E.A., Roca J. (2013). The Nuclear DNA Longevity in Cryopreserved Boar Spermatozoa Assessed Using the Sperm-Sus-Halomax. Theriogenology.

[46] Kuchakulla M., Narasimman M., Khodamoradi K., Khosravizadeh Z., Ramasamy R. (2021). How Defective Spermatogenesis Affects Sperm DNA Integrity. Andrologia.

[47] Woelders H., Matthijs A., Engel B. (1997). Effects of Trehalose and Sucrose, Osmolality of the Freezing Medium, and Cooling Rate on Viability and Intactness of Bull Sperm after Freezing and Thawing. Cryobiology.

[48] Namula Z., Kodama R., Tanihara F., Morita Y., Sato Y., Wittayarat M., Taniguchi M., Otoi T. (2014). Effects of Skim-Milk Supplementation on the Quality and Penetrating Ability of Boar sperm after Long-Term Preservation at 15°C. Acta Vet. Hung..

[49] Gómez-Fernández J., Gómez-Izquierdo E., Tomás C., Mocé E., de Mercado E. (2013). Is Sperm Freezability Related to the Post-Thaw Lipid Peroxidation and the Formation of Reactive Oxygen Species in Boars?. Reprod. Domest. Anim..

[50] Zhang J., Wen C., Zhang H., Duan Y. (2019). Review of Isolation, Structural Properties, Chain Conformation, and Bioactivities of Psyllium Polysaccharides. Int. J. Biol. Macromol..

[51] Prestera T., Talalay P., Alam J., Ahn Y.I., Lee P.J., Choi A.M. (1995). Parallel Induction of Heme Oxygenase-1 and Chemoprotective Phase 2 Enzymes by Electrophiles and Antioxidants: Regulation by Upstream Antioxidant-Responsive Elements (ARE). Mol. Med..

[52] Lin D., Xu C.J., Liu Y., Zhou Y., Xiong S.L., Wu H.C., Deng J., Yi Y.W., Qiao M.F., Xiao H. (2022). Chemical Structures and Antioxidant Activities of Polysaccharides from *Carthamus tinctorius* L.. Polymers.

[53] Weng X.G., Cai M.M., Zhang Y.T., Liu Y., Gao Z.L., Song J., Liu Z.H. (2018). Effect of Astragalus Polysaccharide Addition to Thawed Boar Sperm on in Vitro Fertilization and Embryo Development. Theriogenology.

[54] Zangishhi N., Hajarian H., Karamishabankareh H., Soltani L. (2024). The effect of different concentrations of laminarin on the quality of cryopreserved ram semen. Cryo Lett..

